# Revisiting assumptions in test-negative studies for estimating vaccine effectiveness: the need for a clinical case definition

**DOI:** 10.21203/rs.3.rs-2689147/v1

**Published:** 2023-05-04

**Authors:** Sheena Sullivan, Arseniy Khvorov, Xiaotong Huang, Can Wang, Kylie Ainslie, Joshua Nealon, Bingyi Yang, Benjamin Cowling, Tim Tsang

**Affiliations:** Peter Doherty Institute for Infection and Immunity; Peter Doherty Institute for Infection and Immunity; University of Hong Kong; University of Hong Kong; University of Hong Kong; University of Hong Kong; University of Hong Kong; University of Hong Kong

**Keywords:** Vaccine effectiveness, COVID-19, SARS-CoV-2, study design, test negative design, selection bias, sampling bias

## Abstract

Test negative studies have been used extensively for the estimation of COVID-19 vaccine effectiveness (VE). Such studies are able to estimate VE against medically-attended illness under certain assumptions. Selection bias may be present if the probability of participation is associated with vaccination or COVID-19, but this can be mitigated through use of a clinical case definition to screen patients for eligibility, which increases the likelihood that cases and non-cases come from the same source population. We examined the extent to which this type of bias could harm COVID-19 VE through systematic review and simulation. A systematic review of test-negative studies was re-analysed to identify studies ignoring the need for clinical criteria. Studies using a clinical case definition had a lower pooled VE estimate compared with studies that did not. Simulations varied the probability of selection by case and vaccination status. Positive bias away from the null (i.e., inflated VE consistent with the systematic review) was observed when there was a higher proportion of healthy, vaccinated non-cases, which may occur if a dataset contains many results from asymptomatic screening in settings where vaccination coverage is high. We provide an html tool for researchers to explore site-specific sources of selection bias in their own studies. We recommend all group consider the potential for selection bias in their vaccine effectiveness studies, particularly when using administrative data.

## Introduction

Since the initial roll-out of COVID-19 vaccines, the test-negative design has been frequently applied to enable timely monitoring of COVID-19 vaccine effectiveness (VE).^1^ This design has been extensively used for estimation of influenza VE,^2^ for which studies have often leveraged sentinel surveillance systems where patients presenting with a particular clinical case definition are enrolled from ambulatory or inpatient medical facilities, regardless of their vaccination status, and tested for the pathogen of interest. Those patients testing positive are identified as cases, while those testing negative are identified as non-cases. VE is estimated from the odds ratio comparing the odds of vaccination among the cases versus non-cases.^3,4^ Here, the term “non-cases” is deliberately used because case status is not known at the time of enrolment, and no sampling frame is used to guide recruitment of cases and non-cases, which differentiates the test-negative design from the traditional case-control study.

The test-negative design has been extensively validated for influenza,^3–8^ usually under the scenario described above. We have previously reviewed its application to other pathogens and have cautioned that its suitability needs to be re-examined for each new use.^2^ The applicability of the test-negative design for monitoring COVID-19 VE was not examined until after widespread use and several possible weaknesses were highlighted.^9^

**Figure 1:**
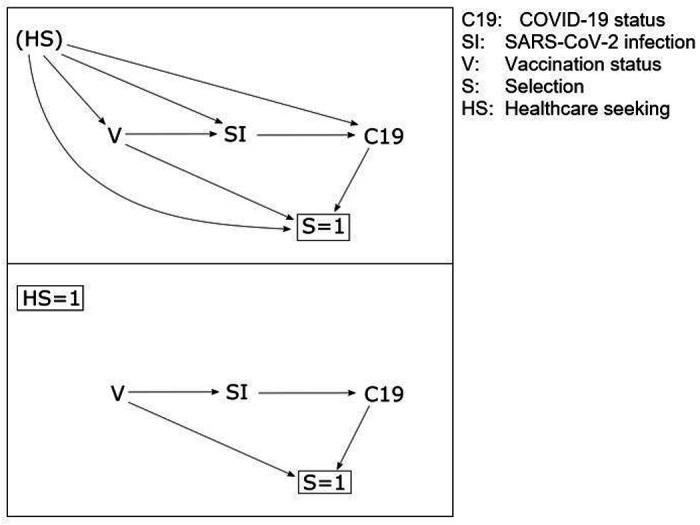
Directed acyclic graph illustrating selection bias in test-negative studies by health-care-seeking behaviour. In the upper panel, health-care-seeking HS confounds the relationship between vaccination V, SARS-CoV-2 infection SI (e.g. by influencing engagement in risk behaviours), and COVID-19 C19 (e.g. because of other healthy behaviours that modify disease severity). Only patients who are tested for SARS-CoV-2 are selected into the study S=1, resulting in collider bias. An individual’s vaccination status V and COVID-19 status C19 influences whether they present for care, are tested and selected into the study. In the lower panel, the test-negative design by restricting participants who meet particular clinical criteria HS=1 presenting to the sentinel site S=1 blocks the biasing paths and enables unbiased estimation of the V-C19 effect.

Here, we focus on one key design feature of the test-negative design that has been variously implemented: the restriction of participants to those meeting a clinical case definition. Prior to COVID-19, laboratory tests for confirmation of infection were typically only conducted on people with clinical symptoms. However, given the pre-symptomatic transmission potential of COVID-19 cases, laboratory tests were conducted on many people without symptoms, so some studies using the test-negative design may include participants that would not meet a clinical case definition. Notwithstanding other sources of bias, the use of a clinical case definition ensures that cases and non-cases are derived from the same source population; i.e. patients who would have presented for care with the disease of interest and been enrolled as cases had they tested positive for the pathogen of interest. The causal model is depicted in Fig. 1.

Clinical restriction underscores two key features of test-negative studies. First, in this design, VE is not estimated against infection per se, but estimates the vaccine’s effectiveness at preventing medically-attended illness (or hospitalised illness, if enrolment is in hospitals). Second, failure to restrict the population in this way breaks the assumption that cases and non-cases are derived from the same source population.^9^ This problem relates to the selection bias that might be induced by differential health seeking between cases and non-cases. ^5,7,9^ Lewnard et al. explored this problem and noted that in scenarios where healthcare seeking is correlated with vaccination, ignoring it inflates VE estimates.^9^

Studies using health services databases may be at greatest risk of this selection bias. These studies typically use data collected for administrative purposes rather than for the study in question. They may assimilate results on a broad range of individuals tested for a variety of reasons. For example, administrative datasets may include a high proportion of people tested asymptomatically as part of screening programs, close contacts tested to clear isolation, or the worried well. The pool of negative test results may be over-represented by people whose degree of risk was associated with their vaccination status (e.g., because their workplace requires both asymptomatic screening and vaccination), which can result in a higher proportion of unvaccinated cases leading to higher VE estimates.

## Evidence from a systematic review

To demonstrate the problem, we explored VE estimates extracted as part of a systematic review^1^ of test-negative design studies that estimated VE against medically attended COVID-19 illness and severe disease (hospitalisation, admission to intensive care unit and/or death) for a primary course of vaccination. Full details are provided elsewhere,^1^ but briefly, papers were included if the authors described the study as a test-negative design or all participants included in the analysis had been tested for SARS-CoV-2, irrespective of clinical criteria. Data were extracted using a standard data collection form, which included whether or not the study used clinical criteria for enrolment.

The search was last updated 11 July 2022 and identified 66 studies that met our inclusion criteria (see supplementary material). Forty-one studies used clinical criteria for enrolment, while 25 did not. Pooled VE was estimated using random effects meta-analysis. VE against medically-attended illness from studies that used clinical criteria was lower (VE: 81%; 95% CI: 78%, 83%) than studies that did not use clinical criteria (VE: 87%; 95% CI: 83%, 90%). On the odds ratio (OR) scale, the ratio of ORs for studies that used clinical criteria versus those that did not was 1.19 (95% CI: 0.96, 1.47). VE against severe disease from studies that used clinical criteria was also lower (VE: 87%; 95% CI: 84%, 90% versus VE: 93%; 95% CI: 91%, 95%), corresponding to a ratio of ORs of 1.57 (95% CI: 1.13, 2.19). At high VE, these inflation factors have a modest impact; however, at lower true VE the impact is great. For example if VE from a study not using clinical case definition were 60%, the VE would be 36% had that study used a clinical case definition.

We note that some studies using administrative data have restricted the study sample to individuals with certain discharge codes to approximate a clinical case definition.^10^ However, discharge diagnoses are assigned after testing, so this approach may still fail to achieve exchangeability between cases and non-cases in terms of their clinical indications for testing. Moreover, such an approach is contingent on assuming that testing was not influenced by the patient’s vaccination status. When test-negative studies are run prospectively, participating providers can be reminded to remain impartial about vaccination status when sampling patients.

## Simulation study

Rapid VE estimation, especially estimation that leverages administrative data and can therefore be done less expensively than studies which follow a sampling framework, is an attractive option. However, research groups and policy makers need to understand the pitfalls of this approach.

The application of a clinical case definition in test-negative studies provides some reassurance that the non-case group reflects the source population of the cases.^12^ While this requirement increases the likelihood that the non-cases have a similar risk of exposure to the virus, it does not guarantee it; i.e., some non-cases may still fail to meet the exposure necessity assumption.^11^ Moreover, the use of clinical criteria seeks to address internal validity; generalizability is limited to the healthcare seeking population.^12^ In some special cases, it may be possible to estimate VE in the whole population; for example, when participants are recruited through point-prevalence surveys^13^ or in studies that limit participants to close contacts of a case such as household transmission studies.^14^ However, those approaches may still suffer from participation bias.^12^

Salvaging internal validity, at a minimum, is important for public health decision making. In VE studies, generalising to the healthcare-seeking population may be satisfactory since it is the burden on our health systems we wish to mitigate with vaccination. Where selection processes fail to ensure the study sample represents the source population, various methods exist to correct the resultant selection bias, but may require additional information unavailable to the researcher.^15–18^ We recommend that all research groups perform an assessment of the degree to which VE is biased under selection scenarios relevant to their setting. The tool we have provided can help with this assessment.

When conducted with a clinical case definition in mind, test-negative studies may be able to provide valid estimates of VE against a specific syndrome of medically-attended disease. When the indications for testing are ignored, the resulting VE is unbiased only when the asymptomatic proportions included into cases and non-cases are the same for the vaccinated and the unvaccinated, which is rare. It is otherwise unclear what the VE estimate represents, but it is unlikely to be a measure of VE against infection, nor medically-attended illness, and is instead some hybrid, the public health implications of which are unclear (and possibly unhelpful). If the goal is to estimate VE against infection, not disease, the test-negative design is not the best design choice, and those choosing it need to fully acknowledge its limitations. The tool we have provided in the supplementary information can help researchers assess the potential for bias under plausible scenarios in their population.

## Conclusions

Rapid VE estimation, especially estimation that leverages administrative data and can therefore be done less expensively than studies which follow a sampling framework, is an attractive option. However, research groups and policy makers need to understand the pitfalls of this approach.

The application of a clinical case definition in test-negative studies provides some reassurance that the non-case group reflects the source population of the cases.^12^ While this requirement increases the likelihood that the non-cases have a similar risk of exposure to the virus, it does not guarantee it; i.e., some non-cases may still fail to meet the exposure necessity assumption.^11^ Moreover, the use of clinical criteria seeks to address internal validity; generalizability is limited to the healthcare seeking population.^12^ In some special cases, it may be possible to estimate VE in the whole population; for example, when participants are recruited through point-prevalence surveys^13^ or in studies that limit participants to close contacts of a case such as household transmission studies.^14^ However, those approaches may still suffer from participation bias.^12^

Salvaging internal validity, at a minimum, is important for public health decision making. In VE studies, generalising to the healthcare-seeking population may be satisfactory since it is the burden on our health systems we wish to mitigate with vaccination. Where selection processes fail to ensure the study sample represents the source population, various methods exist to correct the resultant selection bias, but may require additional information unavailable to the researcher.^15–18^ We recommend that all research groups perform an assessment of the degree to which VE is biased under selection scenarios relevant to their setting. The tool we have provided can help with this assessment.

When conducted with a clinical case definition in mind, test-negative studies may be able to provide valid estimates of VE against a specific syndrome of medically-attended disease. When the indications for testing are ignored, the resulting VE is unbiased only when the asymptomatic proportions included into cases and non-cases are the same for the vaccinated and the unvaccinated, which is rare. It is otherwise unclear what the VE estimate represents, but it is unlikely to be a measure of VE against infection, nor medically-attended illness, and is instead some hybrid, the public health implications of which are unclear (and possibly unhelpful). If the goal is to estimate VE against infection, not disease, the test-negative design is not the best design choice, and those choosing it need to fully acknowledge its limitations. The tool we have provided in the supplementary information can help researchers assess the potential for bias under plausible scenarios in their population.

## Figures and Tables

**Figure 2:**
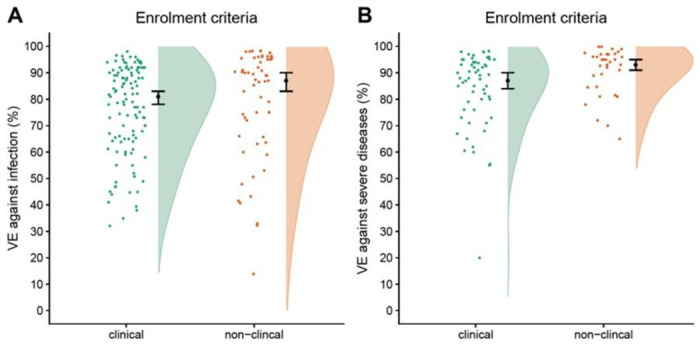
Summary of VE estimates against medically-attended illness (left) and severe disease (right) for 66 studies included in systematic review and published between January 2021 and July 2022. Points indicate the VE point estimate from each study without confidence intervals. Black points with lines show the pooled estimate from the random-effect meta-analysis with 95% confidence intervals. Shaded area is the violin plot, which is the smoothed density of the VE point estimates.

**Figure 3:**
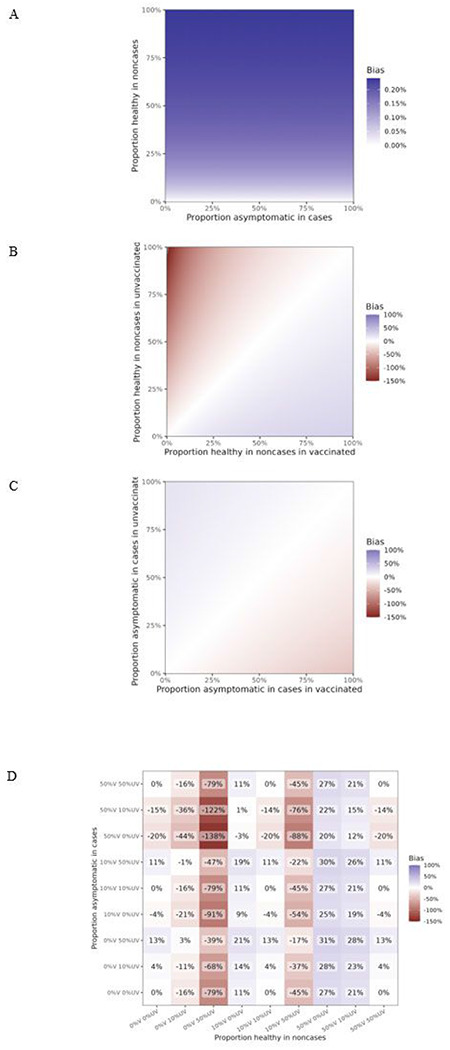
Expected bias (estimated VE minus true VE) in VE estimates from a test-negative study at different values of proportion of asymptomatic (healthy) people who are part of the study as non-cases and proportion of asymptomatically people who are included as cases (proportion is the same for the vaccinated and the unvaccinated). Panel A shows the non-differential case where the proportion asymptomatic is the same for the vaccinated and the unvaccinated, and the bias is minimal (note the scale in the legend). Panels B and C show the bias when the proportion asymptomatic is differential by vaccination status in non-cases (B) and cases (C). The non-differential case is also shown along the diagonal in Panels B and C and while non-zero is negligible and not visible on the plot. Note that for Panel C this is because the proportion of asymptomatic infections among all infections is the same for the vaccinated and the unvaccinated in the simulation under the default parameter set.Panel D shows selected values exploring the bias at different asymptomatic proportions by both vaccination and case status. Axis labels are understood as follows: “25%V 75%UV” indicates that for the vaccinated the proportion asymptomatic is set to 25%, for the unvaccinated it is set to 75%. For all plots, the percent bias indicates the difference in VE estimate compared with the default value of 60%; e.g. a value of −17% means the estimated value is VE=47%. All parameters other than the ones in the X and Y axes are set to their default values as per Table 1.

**Table 1.** Default simulation parameters when VE is unbiased. The parameter “proportion of healthy included as non-cases” for the vaccinated means the proportion of the population (who are vaccinated and healthy) that are included into the study as non-cases. When this parameter is 0, no vaccinated healthy person is included into the study (as is the case in true test-negative studies). When this parameter is at 100%, the entire vaccinated, non-case population is comprised of ‘healthy’ people who are uninfected with the target pathogen (as is the case in case-control studies). Similar logic applies to this parameter for the unvaccinated.

Parameter	Values
Vaccine effectiveness	60%

Proportion of healthy included as non-cases	
Vaccinated	0%
Unvaccinated	0%

Proportion of asymptomatic SARS-CoV 2 infections	
Vaccinated	0%
Unvaccinated	0%

Proportion of asymptomatic included as cases	
Vaccinated	0%
Unvaccinated	0%

Proportion of SARS-CoV 2 infections that are symptomatic	
Vaccinated	50%
Unvaccinated	50%

Risk of SARS-CoV-2 in unvaccinated	1%

Risk SARS-CoV-2 in vaccinated (discounting the effect of vaccination).	1%

Probability of a symptomatic infection with anything other than SARS-CoV-2	20%

Vaccine coverage	70%

## Data Availability

R and html scripts used in simulations are provided with the supplementary material. Papers included in systematic review are listed in the appendix. Any further data extracted from reviewed articles can be provided upon request to Tim K. Tsang timkltsang@gmail.com.

## References

[1] Tsang TK, Sullivan SG, Huang X, Prior infections and effectiveness of SARS-CoV-2 vaccine in test-negative study: A systematic review and meta-analysis. medRxiv 2022: 2022.11.03.22281925. 10.1093/aje/kwae142 PMC11637527 38904437

[2] Chua H, Feng S, Lewnard JA, The Use of Test-negative Controls to Monitor Vaccine Effectiveness: A Systematic Review of Methodology. Epidemiology 2020; 31: 43–64. 31609860 10.1097/EDE.0000000000001116 PMC6888869

[3] Sullivan SG, Feng S, Cowling BJ. Potential of the test-negative design for measuring influenza vaccine effectiveness: a systematic review. Expert Rev Vaccines 2014; 13: 1571–91. 25348015 10.1586/14760584.2014.966695 PMC4277796

[4] Sullivan SG, Tchetgen Tchetgen EJ, Cowling BJ. Theoretical Basis of the Test-Negative Study Design for Assessment of Influenza Vaccine Effectiveness. Am J Epidemiol 2016; 184: 345–53. 27587721 10.1093/aje/kww064 PMC5013887

[5] Ainslie KEC, Haber M, Orenstein WA. Bias of influenza vaccine effectiveness estimates from test-negative studies conducted during an influenza pandemic. Vaccine 2019; 37: 1987–93. 30833155 10.1016/j.vaccine.2019.02.036 PMC6449847

[6] Foppa IM, Haber M, Ferdinands JM, Shay DK. The case test-negative design for studies of the effectiveness of influenza vaccine. Vaccine 2013; 31: 3104–9. 23624093 10.1016/j.vaccine.2013.04.026

[7] Jackson ML, Nelson JC. The test-negative design for estimating influenza vaccine effectiveness. Vaccine 2013; 31: 2165–8. 23499601 10.1016/j.vaccine.2013.02.053

[8] Ainslie KEC, Haber M, Orenstein WA. Challenges in estimating influenza vaccine effectiveness. Expert Rev Vaccines 2019; 18: 615–28. 31116070 10.1080/14760584.2019.1622419 PMC6594904

[9] Lewnard JA, Patel MM, Jewell NR Theoretical Framework for Retrospective Studies of the Effectiveness of SARS-CoV-2 Vaccines. Epidemiology 2021; 32: 508–17. 34001753 10.1097/EDE.0000000000001366 PMC8168935

[10] Thompson MG, Natarajan K, Irving SA, Effectiveness of a Third Dose of mRNA Vaccines Against C0VID-19-Associated Emergency Department and Urgent Care Encounters and Hospitalizations Among Adults During Periods of Delta and Omicron Variant Predominance - VISION Network, 10 States, August 2021-January 2022. MMWR Morb Mortal Wkly Rep 2022; 71: 139–45. 35085224 10.15585/mmwr.mm7104e3 PMC9351525

[11] Stensrud MJ. Identification of Vaccine Effects When Exposure Status Is Unknown. Epidemiology 9900. 10.1097/EDE.0000000000001573 PMC9891279 36696229

[12] Infante-Rivard C, Cusson A. Reflection on modern methods: selection bias–a review of recent developments. International journal of epidemiology 2018; 47: 1714–22. 29982600 10.1093/ije/dyy138

[13] Chadeau-Hyam M, Wang H, Eales O, SARS-CoV-2 infection and vaccine effectiveness in England (REACT-1): a series of cross-sectional random community surveys. The Lancet Respiratory Medicine 2022; 10: 355–66. 35085490 10.1016/S2213-2600(21)00542-7 PMC8786320

[14] Halloran ME, Longini IM Jr., Struchiner CJ. Design and Interpretation of Vaccine Field Studies. Epidemiologic Reviews 1999; 21: 73–88. 10520474 10.1093/oxfordjournals.epirev.a017990

[15] Geneletti S, Mason A, Best N. Adjusting for selection effects in epidemiologic studies: why sensitivity analysis is the only “solution”. Epidemiology 2011; 22: 36–9. 21150353 10.1097/EDE.0b013e3182003276

[16] Geneletti S, Richardson S, Best N. Adjusting for selection bias in retrospective, case–control studies. Biostatistics 2008; 10: 17–31. 18482997 10.1093/biostatistics/kxn010

[17] Greenland S. Basic methods for sensitivity analysis of biases. International journal of epidemiology 1996; 25: 1107–16. 9027513

[18] Westreich D. Berkson’s bias, selection bias, and missing data. Epidemiology 2012; 23: 159–64. 22081062 10.1097/EDE.0b013e31823b6296 PMC3237868

